# Effect of blood pressure on the mortality of the elderly population with (pre)frailty: Results from NHANES 1999–2004

**DOI:** 10.3389/fcvm.2022.919956

**Published:** 2022-08-01

**Authors:** Menghuan Li, Zhenyang Su, Hu Su, Zhi Zuo, Yuan He, Wenming Yao, Jiaming Yang, Kerui Zhang, Hui Wang, Xiangqing Kong

**Affiliations:** ^1^Department of Cardiology, The First Affiliated Hospital of Nanjing Medical University, Nanjing, China; ^2^School of Medicine, Southeast University, Nanjing, China; ^3^Gusu School, Nanjing Medical University, Soochow, China

**Keywords:** blood pressure, elderly, frailty, mortality, association

## Abstract

**Backgrounds:**

The optimal blood pressure of elderly people with frailty or prefrailty is still unclear. We aimed to explore the relationship between blood pressure and mortality in the elderly with (pre)frailty.

**Methods:**

A total of 528 participants aged 60 years and older were exacted for analyses of the association between blood pressure and mortality from the database of the National Health and Nutrition Examination Survey (NHANES) (1999–2004). Kaplan–Meier curves and log-rank tests were used to investigate the differences in survival between groups. Multivariable Cox regression and restricted cubic spline (RCS) analyses were applied to explore the relationship between blood pressure and mortality.

**Results:**

During the median follow-up time of 116.5 [interquartile range (IQR) of 60–186] months, 363 all-cause deaths and 122 cardiac deaths were documented. For all-cause mortality, more participants died with systolic blood pressure (SBP) < 110 mmHg and SBP ≥ 170 mmHg (log-rank *p* = 0.004). After adjusting for confounders, SBP < 110 mmHg [hazard ratio (*HR*) 1.52, 95% *CI*: 0.96–2.41] and SBP ≥ 170 mmHg (*HR* 1.53, 95% *CI*: 1.09–2.15) had higher risks of all-cause mortality compared with SBP within 130–150 mmHg. There were no significant differences in all-cause mortality among DBP categories. A J-curve association was identified between the SBP and hazard ratio for all-cause mortality (*p* for non-linear = 0.028), with 138.6 mmHg as the lowest hazard ratio of all-cause mortality; each 10 mmHg of SBP rise was associated with a 9% increased risk in all-cause mortality (*HR* 1.09, 95% *CI* 1.00–1.18). Additionally, a non-linear relationship was determined between SBP and the hazard ratio for cardiac deaths (*p* for non-linear = 0.030), with 140.1 mmHg as the lowest hazard ratio of cardiac deaths. When SBP was higher than 140.1 mmHg, each 10 mmHg rise in SBP was associated with a 17% increased risk of cardiac deaths (*HR* 1.17, 95% *CI*: 1.02–1.34).

**Conclusion:**

Both lower and higher SBP levels are associated with higher risks of all-cause mortality in older individuals with (pre)frailty. There are J-shaped associations between SBP and mortality, with the optimal SBP being approximately 140 mmHg for this population specifically.

## Introduction

Frailty is a complex condition involving multisystem impairment that decreases the physiology reserve and increases the vulnerability to stress (1). Frailty and prefrailty are quite common with a prevalence of 10.7 and 41.6%, respectively, in an older community-dwelling population (2). Frailty is associated with an increased risk of morbidity and mortality in patients with cardiovascular disease (1, 3–6).

Blood pressure, as well as the prevalence of frailty, rises with age. A few studies assessed the association between blood pressure and mortality in the very old population specifically (5–7). However, there were contradistinctions among these results. Low, as opposed to high, blood pressure was related to a higher risk of mortality in a very old population (5). However, a randomized trial showed that intensive blood pressure lowing therapy resulted in significantly lower rates of fatal and non-fatal major cardiovascular events and all-cause death in elderly people (6). Odden et al. demonstrated that increased blood pressure was associated with a lower rate of death in frail persons defined by walk speed (7).

The optimal blood pressure is still unclear in elderly people with frailty or prefrailty. Therefore, we conducted an observational study that explores the relationship between blood pressure in the elderly with frailty and mortality.

## Methods

### Study population

The National Health and Nutrition Examination Survey (NHANES) recruited a representative sample of participants from the United States population based on a complex, multistage probability design (8). The program collected data from demographic, clinical, and laboratory tests for surveyed individuals. We analyzed the data from 1999 to 2004 including 29,608 participants. Of those, 6,210 participants were available for the items of frailty. Consequently, after excluding those with missing data on mortality (*n* = 4,281), non-(pre)frailty (*n* = 799), and an age < 60 years (*n* = 271), a total of 528 subjects were enrolled in further analysis. The survey protocol was approved by the National Center for Health Statistics (NCHS) research ethics review board, and all participants provided written informed consent.

### Assessment of (pre)frailty

The frailty was evaluated by the Fried phenotype model, which was the most cited and generally regarded as the standard model of frailty (9). We used an adapted version of the original Cardiovascular Health Study model, considering the four items below: (1) unintentional weight loss; (2) exhaustion; (3) weakness; and (4) low physical activity.

There were four criteria available from the NHANES database. (1) Unintentional weight loss was defined as low body mass index (BMI < 18.5 kg/m^2^). (2) Exhaustion was evaluated by the following question: “do you have difficulty walking between rooms?” An answer of “yes” results in one point to frailty. (3) Weakness was determined by answering “yes” to the question “do you have difficulty lifting or carrying 10lbs?” (4) Low physical activity was recorded by self-reported reduced physical activity compared with others of the same age. Individuals who met three or more criteria were classified as frailty, and individuals who met one or two criteria were classified as prefrailty.

### Mortality data

The data on mortality was obtained from a probabilistic match between the NHANES and the National Death Index (NDI), which provided follow-up information from the date of survey participation through 31 December 2015 (10). The underlying cause of death was recorded based on the International Classification of Disease, Tenth Revision (ICD-10) (11). The current study used the data on all-cause mortality and cardiac death.

### Covariates

Demographic data, such as age, gender, race/ethnicity, and education level were collected from self-reports. Comorbidities, such as diabetes, hyperlipidemia, chronic obstructive pulmonary disease (COPD), stroke, coronary artery disease, asthma, cancer, thyroid disease, and chronic kidney disease were also obtained from self-reports. Smokers (those answering “every day” or “some days” to the question “do you now smoke cigarettes?”) and alcohol users (those ingesting at least 12 alcoholic drinks per year) were documented using questionnaire data. Systolic blood pressure (SBP) and diastolic blood pressure (DBP) were determined using the average values of three consecutive blood pressure readings after resting quietly for 5 min.

### Statistical analysis

Data distribution was determined by using the histogram and Q-Q-Plot. Non-normal distributed continuous data were reported as median with interquartile range (IQR) and compared using a non-parametric Mann–Whitney *U* test. Categorical variables were calculated with counts and percentages and were compared using the chi-square test. SBP was divided into five groups (<110, 110–130, 130–150, 150–170, and ≥170 mmHg). Similarly, DBP was also stratified into five categories (<60, 60–70, 70–80, 80–90, and ≥90 mmHg). The ranges set took into account the limit and interval of blood pressure control.

The differences in cumulative survival rates according to SBP and DBP categories were analyzed by Kaplan–Meier survival curves and the log-rank test. Multivariable Cox regression models were used to evaluate the independent association of blood pressure with all-cause and cardiac mortality. We included covariates in the multivariable model when variables showed *p* < 0.05 at univariable analysis or variables had a clinical relationship with mortality. Age, gender, race, diabetes, stroke, coronary artery disease, smoker, frailty, and antihypertensive medication were included in the multivariable model. A restricted cubic spline (RCS) regression fitted with a Cox regression model [with three knots located at 10^th^, 50^th^, and 90^th^ percentiles confirmed according to the Akaike information criterion (AIC) (12)] was applied to explore any nonlinear relationship between blood pressure and mortality. The inflection point of RCS was confirmed by finding the blood pressure value corresponding to the lowest point of the hazard ratio (*HR*). All statistical analyses were performed by R software (version 4.0.5, The R Foundation for Statistical Computing) and SPSS software (version 25). A two-sided *p* < 0.05 was considered statistically significant.

## Results

### Baseline characteristics

Of the 528 participants, 40.5% were male, most of them were non-Hispanic white ethnics, 24.8% had a diabetes history, 11.2% had a stroke history, and 16.3% were identified as frailty. There were 56 (65.1%) frail participants aged lower than 75 years. The median age with the interquartile range of overall participants was 70 [65–78] years, SBP was 138 [125–158] mmHg, and DBP was 71 [63–77] mmHg (Table 1). Participants in the dead group were older than those in the alive group [73 (66–80) vs. 66 (63–70) years, *p* < 0.001]. The proportion of men and those displaying diabetes, stroke, exhaustion, low BMI, and frailty was significantly higher in participants who died compared with those who were alive (all *p* < 0.05). There were no significant differences in other variables.

**Table 1:** Baseline characteristics of the analyzed population.

**Factors**	**Overall** ***N*** = **528**	**Dead** ***N1*** = **363**	**Alive** ***N2*** = **165**	***P*** **-value**
Age (years), median [Q1–Q3]	70 [65–78]	73 [66–80]	66 [63–70]	<0.001
Male, *n* (%)	214 (40.5)	159 (43.8)	55 (33.3)	0.023
Race/ethnicity, *n* (%)				0.003
Mexican American	149 (28.2)	88 (24.2)	61 (37)	
Other Hispanic	31 (5.9)	16 (4.4)	15 (9.1)	
Non-Hispanic white	228 (43.2)	169 (46.6)	59 (35.8)	
Non-Hispanic Black	104 (19.7)	79 (21.8)	25 (15.2)	
Other race	16 (3.0)	11 (2.0)	5 (3.0)	
Education level, *n* (%)				0.518
Less than high school	314 (59.5)	215 (59.2)	99 (60.0)	
High school	100 (18.9)	73 (20.1)	27 (16.4)	
More than high school	114 (21.6)	75 (20.7)	39 (23.6)	
SBP (mmHg), median [Q1–Q3]	138 [125–158]	141 [126–161]	136 [124–152]	0.030
SBP categories, *n* (%)				0.096
<110 mmHg	34	24 (6.6)	10 (6.1)	
110–130mmHg	140	94 (25.9)	46 (27.9)	
130–150mmHg	165	106 (29.2)	59 (35.8)	
150–170mmHg	116	79 (21.8)	37 (22.4)	
≥170mmHg	73	60 (16.5)	13 (7.9)	
DBP (mmHg), median [Q1–Q3]	71 [63–77]	71 [63–76]	71 [64–78]	0.103
DBP categories, *n* (%)				0.380
<60 mmHg	92 (17.4)	69 (19.0)	23 (13.9)	
60–70 mmHg	149 (29.2)	106 (29.2)	43 (26.1)	
70–80 mmHg	192 (36.4)	126 (34.7)	66 (40.0)	
80–90 mmHg	70 (13.3)	44 (12.1)	26 (15.8)	
≥90 mmHg	25 (4.7)	18 (5.0)	7 (4.2)	
Comorbidities, *n* (%)				
Diabetes	131 (24.8)	102 (28.1)	29 (17.6)	0.009
Hyperlipidemia	252 (47.7)	166 (45.7)	86 (52.1)	0.173
COPD	66 (12.5)	47 (12.9)	19 (11.5)	0.645
Stroke	59 (11.2)	52 (14.3)	7 (4.2)	0.001
CAD,	74 (14%)	58 (16.0)	16 (9.7)	0.054
Asthma	71 (13.4)	44 (12.1)	27 (16.4)	0.185
Cancer	88 (16.7)	67 (18.5)	21 (12.7)	0.102
Thyroid disease	376 (71.2)	253 (69.7)	123 (74.5)	0.254
CKD	5 (0.9)	4 (1.1)	1 (0.6)	1.000
Smoker, *n* (%)	159 (30.1)	124 (34.2)	35 (21.2)	0.003
Alcohol user, *n* (%)	293 (55.5)	208 (57.3)	85 (51.5)	0.215
Antihypertensive medication, *n* (%)	506 (95.8)	350 (96.4)	156 (94.5)	0.318
Frailty index, *n* (%)				
Exhaustion	159 (30.1)	126 (34.7)	33 (20.0)	0.001
Low physical activity	224 (42.4)	160 (44.1)	64 (38.8)	0.254
Weakness	421 (79.7)	288 (79.3)	133 (80.6)	0.737
Low BMI	20 (3.8)	19 (5.2)	1 (0.6)	0.005
Frail status, *n* (%)				0.003
Frailty	86 (16.3)	71 (19.6)	15 (9.1)	
Prefrailty	442 (83.7)	292 (80.4)	150 (90.9)	

Q1, quartile 1; Q3, quartile 3; COPD, chronic obstructive pulmonary disease; CAD, coronary artery disease; CKD, chronic kidney disease; BMI, body mass index.

### Association between blood pressure and mortality

**Figure 1:**
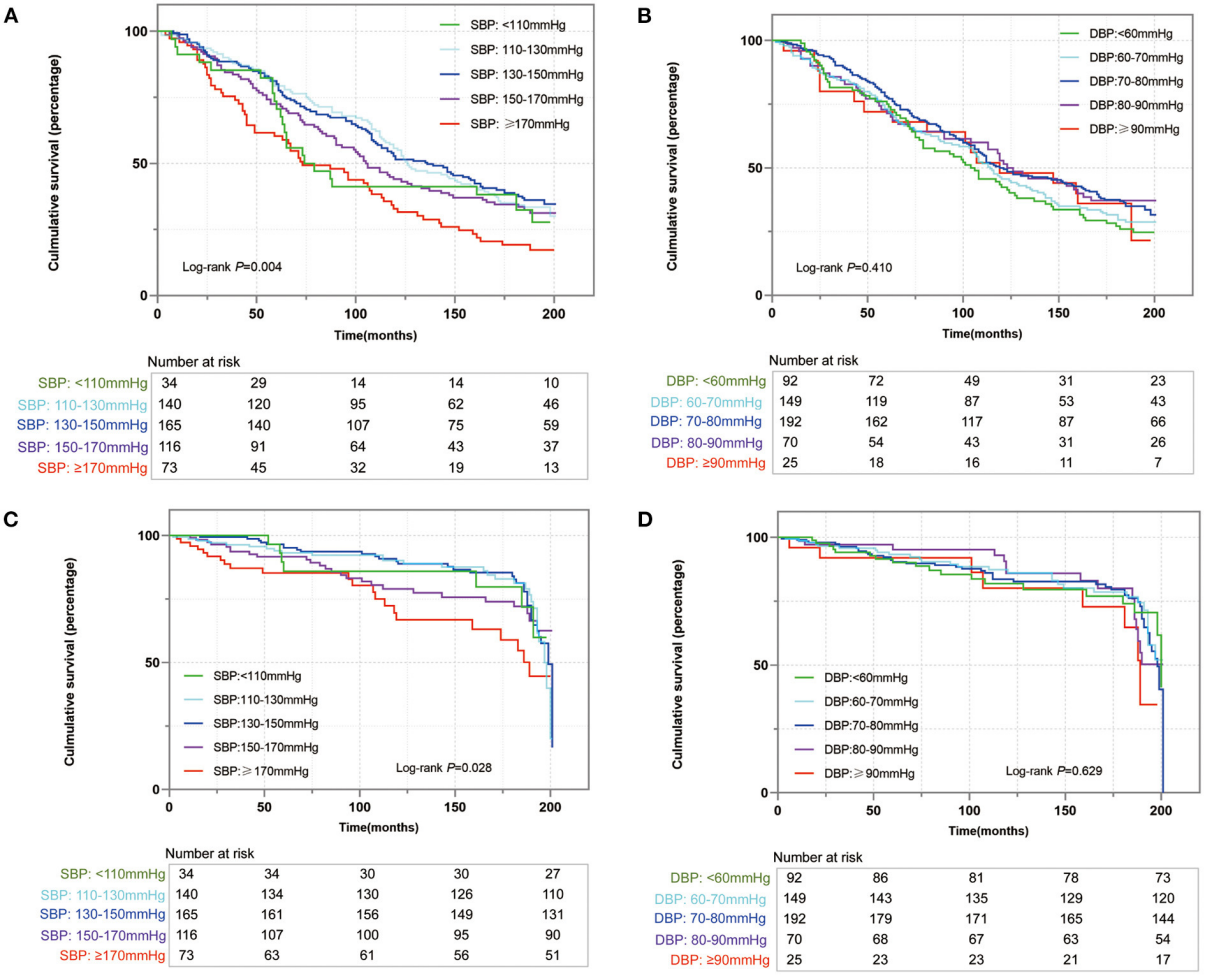
The Kaplan–Meier analysis of all-cause mortality **(A)** and cardiac mortality **(C)** for systolic blood pressure (SBP), and all-cause mortality **(B)** and cardiac mortality **(D)** for diastolic blood pressure (DBP).

**Figure 2:**
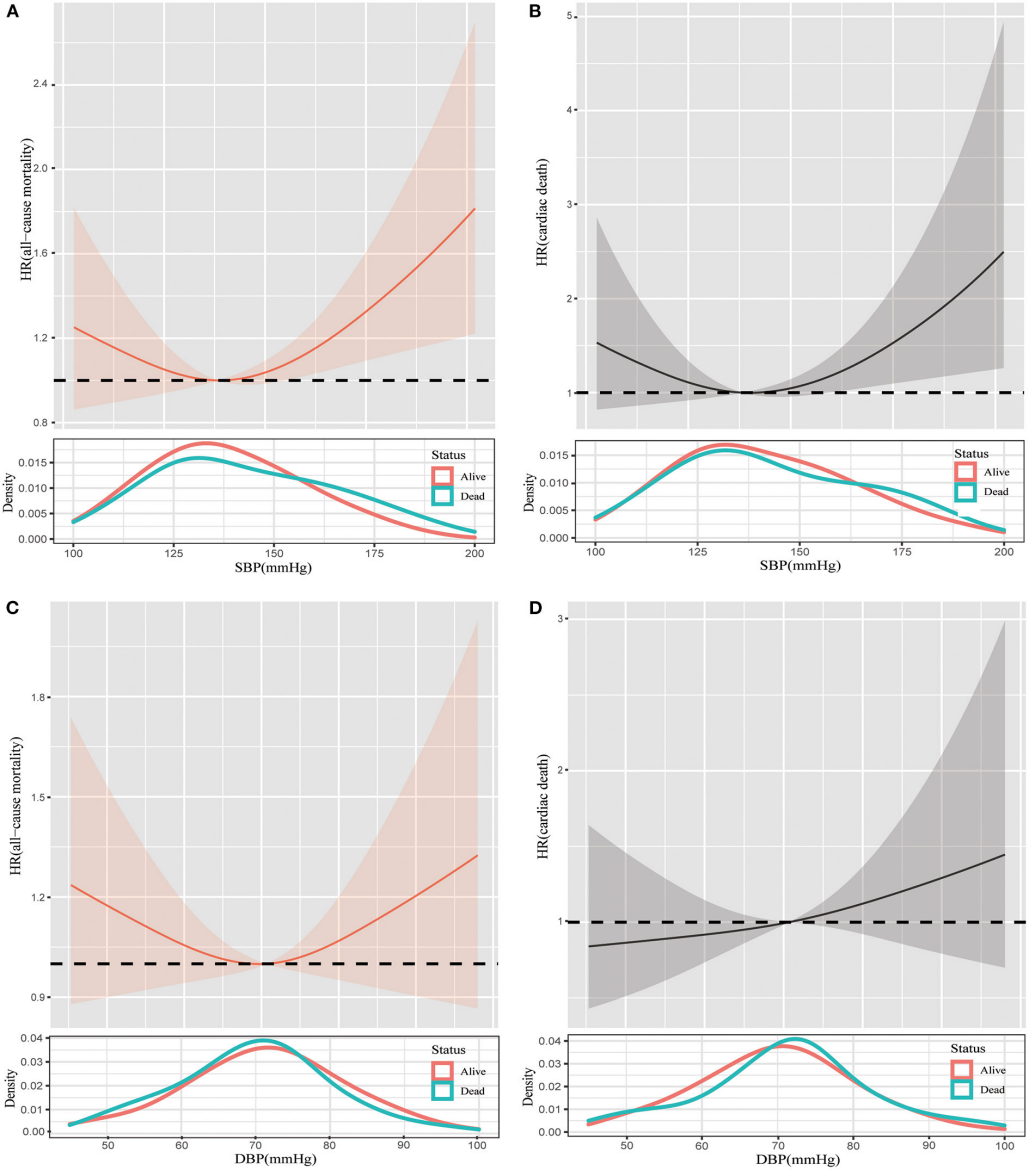
Restricted cubic spline plots of the association between SBP and all-cause mortality **(A)**, SBP and cardiac death **(B)**, DBP and all-cause mortality **(C)**, and DBP and cardiac death **(D)** in (pre)frail participants. Analysis was adjusted for age, gender, race, diabetes, stroke, coronary artery disease, smoker, antihypertensive medication, and frailty. SBP, systolic blood pressure; DBP, diastolic blood pressure; HR, hazard ratio.

During the median follow-up time of 116.5 (IQR: 60–186) months, 363 all-cause deaths were documented. As shown in Figure 1A, for all-cause mortality, more participants died with SBP < 110 mmHg and with SBP ≥ 170 mmHg (log-rank *p* = 0.004). Individuals with SBP within 130–150 mmHg had the lowest cumulative percentages of mortality compared with other SBP categories (Figures 1A,C, all log-rank *p* < 0.05). However, no significant differences were observed in mortality among DBP categories (Figures 1B,D). After adjusting for age, gender, race, diabetes, stroke, CAD, smoker, antihypertensive medication, and frailty, the risks of all-cause mortality were all higher in SBP categories at both sides of 130–150 mmHg, it was shown that SBP ≥ 170 mmHg had a 1.53-fold risk of all-cause mortality compared with SBP within 130–150 mmHg, and SBP ≤ 110 mmHg had 52% higher risks than SBP within 130–150 mmHg (Table 2). Furthermore, the restricted cubic spline analysis revealed that a J-curve association was identified between SBP and hazard ratio for all-cause mortality (*p* for non-linear = 0.028, Figure 2A). Consequently, we identified an inflection point of 138.6 mmHg for SBP as the lowest hazard ratio of all-cause mortality. The risk of all-cause mortality tended to increase with the increase of SBP, indicating that when SBP was higher than 138.6 mmHg, each 10 mmHg of SBP rise was associated with a 9% increased risk in all-cause mortality (*HR* 1.09, 95% *CI*: 1.00–1.18) (Table 3). In addition, the association between DBP and hazard ratio for all-cause mortality was linear (*p* for non-linear > 0.05, Figure 2C), even though the shape of the spline was concave.

**Table 2:** Cox regression analyses of all-cause mortality for SBP.

**Categories**	**Deaths/** **All individuals**	**Unadjusted model**		**Adjusted model**	
		**HR (95% CI)**	***P*** **-value**	**HR (95% CI)**	***P*** **-value**
Age (years)		**1.08 (1.06–1.10)**	**<0.001**	**1.09 (1.07–1.11)**	**<0.001**
Gender					
Male	159/214	Ref.		Ref.	
Female	204/314	0.84 (0.65–1.08)	0.165	**0.72 (0.54–0.96)**	**0.024**
Race					
Mexican American	88/149	Ref.			
Other Hispanic	16/31	0.68 (0.36–1.29)	0.242	0.68 (0.40–1.18)	0.168
Non-Hispanic white	169/228	**1.42 (1.04–1.93)**	**0.026**	1.11 (0.84–1.47)	0.463
Non-Hispanic Black	79/104	1.43 (0.98–2.07)	0.061	1.23 (0.90–1.69)	0.196
Other race	11/16	1.17 (0.58–2.35)	0.658	0.76 (0.40–1.46)	0.414
Diabetes					
Yes	102/131	**1.39 (1.05–1.83)**	**0.020**	**1.62 (1.19–2.21)**	**0.002**
No	261/397	**Ref**.		**Ref**.	
Stroke					
Yes	52/59	1.94 (1.39–2.72)	**<0.001**	1.34 (0.98–1.83)	0.070
No	311/469	**Ref**.		Ref.	
CAD					
Yes	58/74	1.12 (0.79–1.58)	0.524	1.10 (0.82–1.47)	0.541
No	305/454	Ref.		Ref.	
Smoker					
Yes	206/287	1.22 (0.95–1.57)	0.120	**1.40 (1.06–1.84)**	**0.014**
No	157/241	Ref.		**Ref**.	
Frail status					
Prefrailty	292/442	**Ref**.		**Ref**.	
Frailty	71/86	**1.92 (1.36–2.72)**	**<0.001**	**1.80 (1.36–2.40)**	**<0.001**
Antihypertensive medication					
Yes	350/506	1.45 (0.79–2.65)	0.232	1.13 (0.63–2.03)	0.688
No	13/22	Ref.		Ref.	
SBP categories					
<110 mmHg	24/34	1.30 (0.83–2.03)	0.246	1.52 (0.96–2.41)	0.074
110–130 mmHg	94/140	1.04 (0.79–1.37)	0.799	1.02 (0.77–1.36)	0.745
130–150 mmHg	106/165	Ref.		Ref.	
150–170 mmHg	79/116	1.19 (0.89–1.59)	0.254	1.00 (0.74–1.36)	0.992
≥170 mmHg	60/73	**1.78 (1.30–2.45)**	**<0.001**	**1.53 (1.09–2.15)**	**0.015**

The analyses were adjusted for age, gender, race, diabetes, stroke, coronary artery disease, smoker, antihypertensive medication, and frailty. SBP, systolic blood pressure; CAD, coronary artery disease; Ref, reference. HR, hazard ratio; CI, confidence interval. The bold value indicates a statistical *p* value < 0.05.

**Table 3:** The threshold effect analysis of SBP on mortality using a piecewise Cox regression model.

	**Inflection point**	**Groups**	**HR (95% CI)**	***P*** **-value**
All–cause mortality (each unit = 10 mmHg)	138.6 mmHg	≤138.6 mmHg	0.89 (0.76–1.03)	0.885
		>138.6 mmHg	1.09 (1.00–1.18)	0.046
Cardiac death (each unit = 10 mmHg)	140.1 mmHg	≤140.1 mmHg	0.94 (0.73–1.21)	0.632
		>140.1 mmHg	1.17 (1.02–1.34)	0.030

The analyses were adjusted for age, gender, race, diabetes, stroke, coronary artery disease, smoker, antihypertensive medication, and frailty. SBP, systolic blood pressure; HR, hazard ratio; CI, confidence interval.

A total of 122 cardiac deaths were observed. There were no statistical differences in the rate of cardiac deaths among blood pressures, regardless of SBP and DBP (Figures 1C,D). However, a non-linear relationship was determined between SBP and hazard ratio for cardiac death (*p* for non-linear = 0.030, Figure 2B). Table 3 shows that 140.1 mmHg was the threshold of SBP with the lowest hazard ratio for cardiac deaths. When SBP was higher than 140.1 mmHg, each 10 mmHg rise was associated with a 17% increased risk of cardiac deaths (*HR* 1.17, 95% *CI*: 1.02–1.34). Similarly, a linear relationship was detected between DBP and hazard ratio for cardiac deaths (*p* for non-linear > 0.05, Figure 2D).

### Non-frailty, blood pressure, and mortality

To explore the associations between blood pressure and mortality in elderly participants with non-frailty, a total of 1,001 older non-frail participants were extracted from the same dataset of the NHANES. Compared with individuals without frailty, individuals with frailty had more diabetes, chronic obstructive pulmonary disease (COPD), stroke, CAD, asthma, and thyroid disease (Supplementary Table S1, all *p* < 0.05). Participants without frailty had much higher education levels than those with (pre)frailty. There were more deaths in participants with prefrailty and frailty than those in participants with no-frailty. Furthermore, Cox regression analyses showed that participants with frailty had a 3.2-fold risk in all-cause mortality compared with those with no-frailty, and the prefrail population had 70% higher risks of all-cause mortality compared with a non-frail population (Supplementary Table S2). Moreover, we drew splines to assess the changes in hazard ratios across blood pressure (Supplementary Figure S1) in non-frail people. There were tendencies that higher blood pressure, regardless of SBP and DBP, had increased risks of all-cause mortality and cardiac death.

### Subgroup analyses

**Figure 3:**
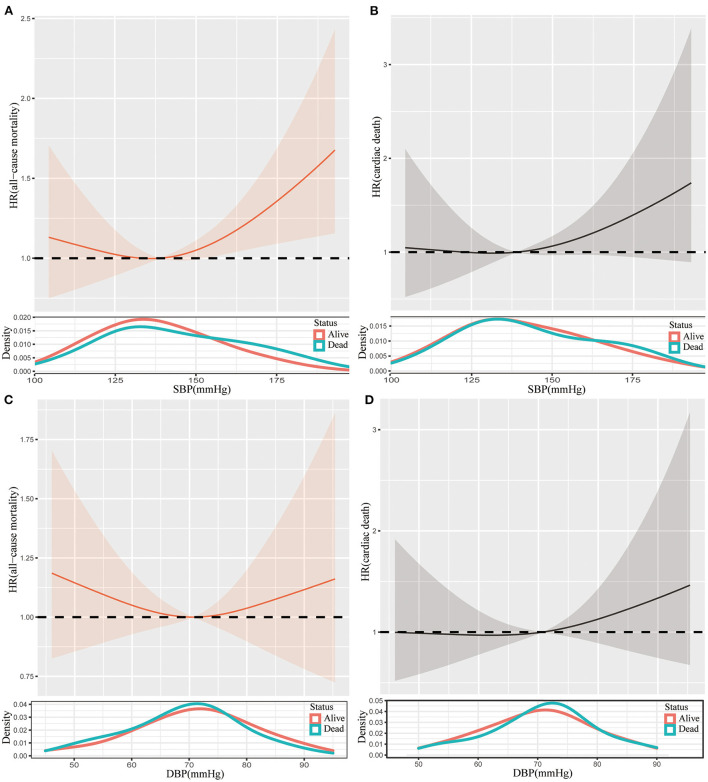
Restricted cubic spline plots of the association between SBP and all-cause mortality **(A)**, SBP and cardiac death **(B)**, DBP and all-cause mortality **(C)**, and DBP and cardiac death **(D)** in prefrail participants. Analysis was adjusted for age, gender, race, diabetes, stroke, coronary artery disease, smoker, antihypertensive medication. SBP, systolic blood pressure; DBP, diastolic blood pressure; HR, hazard ratio.

We conducted subgroup analyses stratified by the frail status (frailty, prefrail, and non-frailty) and age (<75 and ≥75 years). As shown in Supplementary Table S3, SBP <110 mmHg and ≥170 mmHg had higher risks of all-cause mortality compared with SBP within 130–150 mmHg in prefrail individuals. However, there were no differences in the risks of cardiac death across SBP categories (Supplementary Table S4). Splines illustrated that the risks of all-cause mortality initially decreased with the increase of SBP in participants with prefrailty, and then the risks increased when SBP was higher than the inflection point (Figure 3A). A similar trend was presented in the association between DBP and the risk of all-cause mortality (Figure 3C). While the associations between mortality risks and blood pressure (both SBP and DBP) tended to be linear (Figures 3B,D).

**Figure 4:**
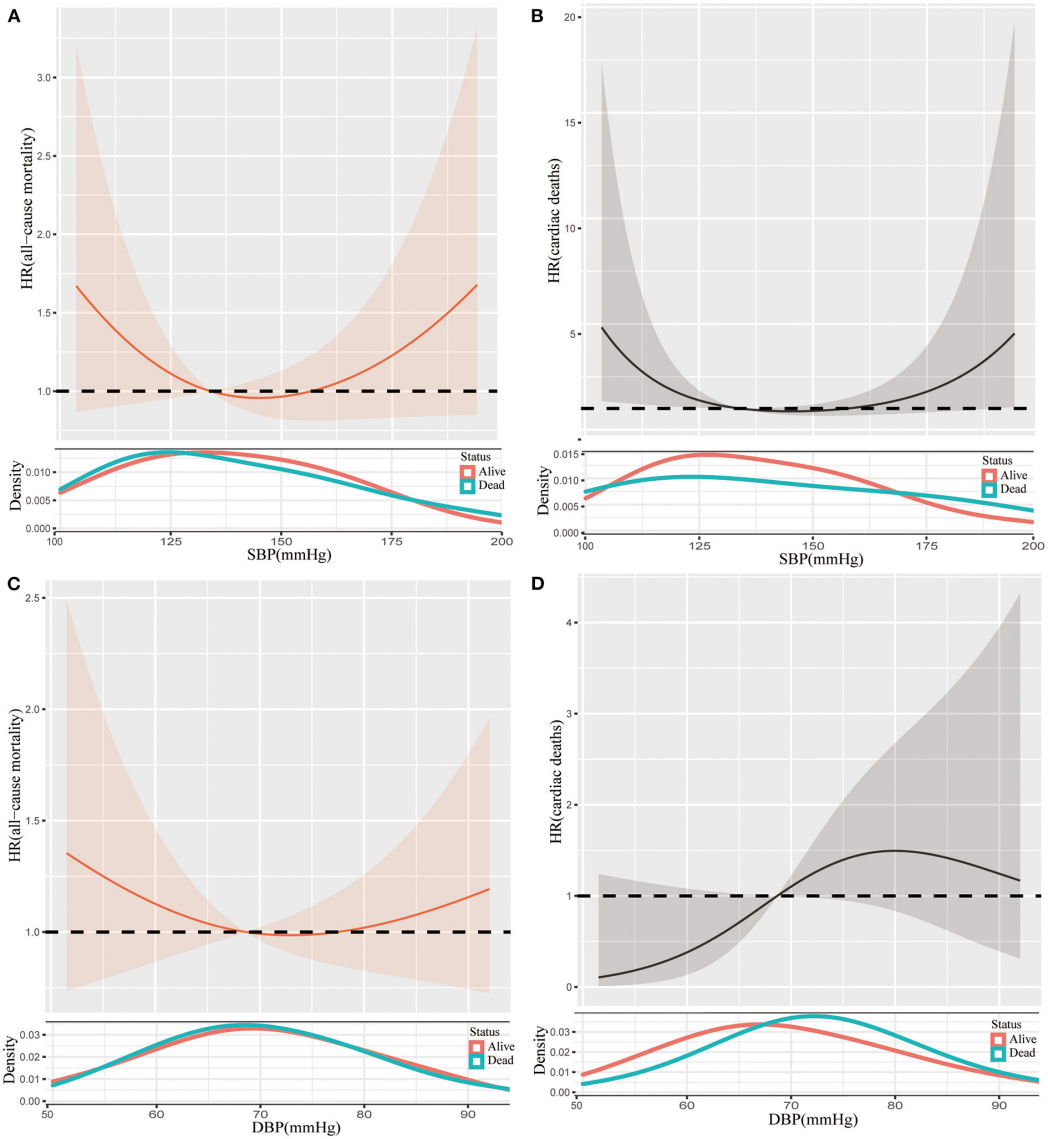
Restricted cubic spline plots of the association between SBP and all-cause mortality **(A)**, SBP and cardiac death **(B)**, DBP and all-cause mortality **(C)**, and DBP and cardiac death **(D)** in frail participants. Analysis was adjusted for age, gender, race, diabetes, stroke, coronary artery disease, smoker, antihypertensive medication. SBP, systolic blood pressure; DBP, diastolic blood pressure; HR, hazard ratio.

As for those with frailty, we could also observe J-shape splines of the association between all-cause mortality risks and blood pressure, but the descending splines to the left of the inflection point were steeper than those in prefrail individuals (Figures 4A,C). Notably, the U-shaped splines of the association between SBP and risks of cardiac death (Figure 4B). Interestingly, a convex spline was determined for the association between DBP and risks of cardiac death (Figure 4D).

For participants aged <75 years, there were no differences in the risks of all-cause mortality across five categories (Supplementary Table S3). In addition, there were J-shape associations between blood pressure and all-cause mortality regardless of SBP and DBP (Supplementary Figures S2A,C), but the spline was smooth in terms of the association between SBP and risk of cardiac death (Supplementary Figure S2B). Similarly, the relationship between DBP and cardiac death was a convex curve (Supplementary Figure S2D).

Compared with SBP within 130–150 mmHg, other categories of SBP had higher risks of all-cause mortality in participants who were older than 75 years (Supplementary Table S3). When we analyzed the association between SBP and cardiac deaths, SBP within 130–150 mmHg retained lower risks of cardiac death among other SBP categories (Supplementary Table S4). Irrespective of SBP and DBP, or risks of all-cause mortality and cardiac death, the associations between them showed J-shape splines (Supplementary Figure S3).

## Discussion

This study resulted in four main findings. First, we found that elderly people with (pre)frailty with SBP below 110 mmHg had higher all-cause mortality compared with those whose SBP was above 110 mmHg in an unadjusted analysis. Second, when fully adjusting confounders, participants with SBP below 110 mmHg remained significantly associated with greater mortality. Third, J-curve associations were identified between SBP and all-cause mortality and cardiac deaths. Finally, each 10 mmHg increase in SBP increased the risk of cardiac deaths by 17% when SBP was higher than 140.1 mmHg.

The 2018 guidelines of the European Society of Cardiology (ESC) set the goal of SBP of 130–140 mmHg for individuals aged ≥ 65 years (13). While the 2017 American College of Cardiology guidelines recommends the target range of SBP < 130 mmHg for people aged ≤ 65years (14). The optimal SBP was still unclear in people with frailty (15). Kremer et al. discussed the impact of SBP on mortality in participants with frailty and without frailty. They concluded that there was a possible protective effect of increased SBP in frail older adults regarding all-cause mortality. In addition, they showed a tendency toward lower risk among frail participants with SBP ≥ 130 mmHg (15). However, in our study, we presented a J-shaped association between SBP and all-cause mortality, illustrating a tendency toward higher risk for older (pre)frail individuals with SBP > 138.6 mmHg. When we split the participants into frailty and prefrailty further, there were also J-shaped associations between SBP and all-cause mortality in both frail and prefrail individuals.

Frailty or prefrailty is correlated with aging greatly (2). It was pointed out that blood-pressure-lowering therapy was one of the few interventions to reduce the risk of mortality in frail older individuals (1). However, there were contradictory results that emphasize the role of blood pressure in mortality for older patients with hypertension. Several studies showed that lower blood pressure level in old age was associated with better survival (6, 16). The SPRINT trial randomly recruited patients 75 years or older into intensive treatment (target SBP < 120 mmHg) and standard treatment (target SBP < 140 mmHg), showing that intensive treatment was associated with lower rates of fatal and nonfatal major cardiovascular events and death from all causes (6). Several studies demonstrated that lower blood pressure correlated well with higher mortality in the elderly population (5, 11). The results above merely represented the very old population, which did not include individuals younger than 75 years. In our study, we included individuals 60 years or older to explore the impact of blood pressure on mortality. Consistent with some previous results, we found that lower SBP levels contributed to higher all-cause mortality. However, an association between blood pressure and cardiac death could not be shown in unadjusted analyses. The subgroup analysis in our study also demonstrated that this association was more sensitive in participants aged ≥75 years. Additionally, as the splines corresponded to both risk and blood pressure, too high blood pressure was also associated with a higher risk of all-cause mortality whether participants were older than 75 years or not.

Previous studies indicated that the reverse relationship between blood pressure and mortality is due to a failure to adjust confounders, such as current health status and frailty (16–18). Age itself was an independent predictor of mortality regardless of blood pressure and health status (19, 20). We attempted to identify related confounders to draw a more reliable relationship between blood pressure and prognosis. After adjusting for age, health status, and other covariates, the lower level of SBP remained the independent predictor of worse outcomes in terms of all-cause mortality.

In our study, we mainly discussed the impact of blood pressure on frail older people. A prospective cohort study showed that the frail index was associated with all-cause mortality and cause-specific mortality independent of chronological age in younger and older Chinese adults (21), which meant frailty or prefrailty occurred at a quite young age. Therefore, we set the age range at 60 years or older in our study. The SBP level was still an independent factor that affected the outcome of frail or prefrail individuals. As for non-frail participants, the risks of mortality increased with the elevation of blood pressure. But for the participants with frailty or prefrailty, J-shape splines were determined regarding the association between SBP and all-cause mortality. Neither lower nor higher blood pressures were beneficial.

A population-based cohort study determined a U-shaped association between mortality and SBP, with 164.2 mmHg being related to the lowest rate of mortality (5). This study only revealed the threshold of SBP in people aged 85 years or older. There was a tendency toward J-curve mortality risk in the present study. The lowest J-points for all-cause morality were 138.6 and 140.1 mmHg for cardiac deaths, respectively. Both thresholds were approximately 140 mmHg, indicating that SBP far from J-point was associated with higher risks of all-cause mortality and cardiac deaths. A study concluded that, for men aged 85 years and older, SBP of approximately 182 mmHg had lower mortality compared with SBP of approximately 134 mmHg for men aged 65–84 years (14). Combining previous results and those of the current study, an optimal SBP of approximately 140 mmHg for people aged 60 years and older may be reasonable, which accords with the recommendation of the target SBP (14).

Since J-shaped associations between SBP and mortality were detected, we used two piecewise Cox regression analyses to examine the effect of blood pressure on mortality from two sides. SBP higher than 138.6 mmHg was associated with a higher risk of all-cause mortality, indicating that each 10 mmHg increase in SBP led to a 9% risk of all-cause of mortality. Meanwhile, SBP higher than 140 mmHg was also significantly associated with a higher rate of cardiac death. Each 10 mmHg increment in SBP contributed to a 17% risk of cardiac deaths. This emphasized that the most optimal point of SBP control could be approximately 140 mmHg.

There are some limitations to the present study. First, the blood pressure analyzed in this study was measured at just one point in time; it could not reflect the long-term blood pressure control. Furthermore, the specific type of hypertensive medication was unclear in this study, which might influence the findings to some extent. Additionally, the sample of frail individuals was small, which may bias the results of this study. Moreover, the definition of frailty was modified in accordance with the available data of the NHANES. Although such a method was well adapted in other literature, we are still worried about the potential deviation from the standard model. Lastly, the results of the current study were derived from the population of the United States; inferences to any other regions or populations should be cautious.

## Conclusion

Lower SBP levels are associated with higher all-cause mortality in older individuals with (pre)frailty. There are J-shaped associations between SBP and mortality, with the optimal SBP of approximately 140 mmHg for this population specifically.

## Data availability statement

Publicly available datasets were analyzed in this study. This data can be found at: https://www.cdc.gov/nchs/nhanes/.

## Ethics statement

The studies involving human participants were reviewed and approved by NCHS research ethics review board. The patients/participants provided their written informed consent to participate in this study.

## Author contributions

ML: conceptualization, methodology, investigation, formal analysis, and writing-original draft. ZS and HS: formal analysis and writing-review and editing. ZZ, YH, and WY: writing-review and editing. JY and KZ: data curation. XK: validation and supervision. HW: conceptualization and investigation. All authors contributed to the article and approved the submitted version.

## Funding

This paper was funded by the Science Foundation of Gusu School (GSKY20210105) and the Natural Science Foundation of Jiangsu Province (BK2012648). The funding body played no role in the design, writing, or decision to publish this paper.

## Conflict of interest

The authors declare that the research was conducted in the absence of any commercial or financial relationships that could be construed as a potential conflict of interest.

## Publisher's note

All claims expressed in this article are solely those of the authors and do not necessarily represent those of their affiliated organizations, or those of the publisher, the editors and the reviewers. Any product that may be evaluated in this article, or claim that may be made by its manufacturer, is not guaranteed or endorsed by the publisher.
