# Supplementary material for: Effect of blood pressure on the mortality of the elderly population with (pre)frailty: Results from NHANES 1999–2004
Source: Front Cardiovasc Med. 2022 Aug 1;9:919956. doi: 10.3389/fcvm.2022.919956 (PMC9376324; doi:10.3389/fcvm.2022.919956)
Supplement: Supplementary file 1 [file Data_Sheet_1.pdf]

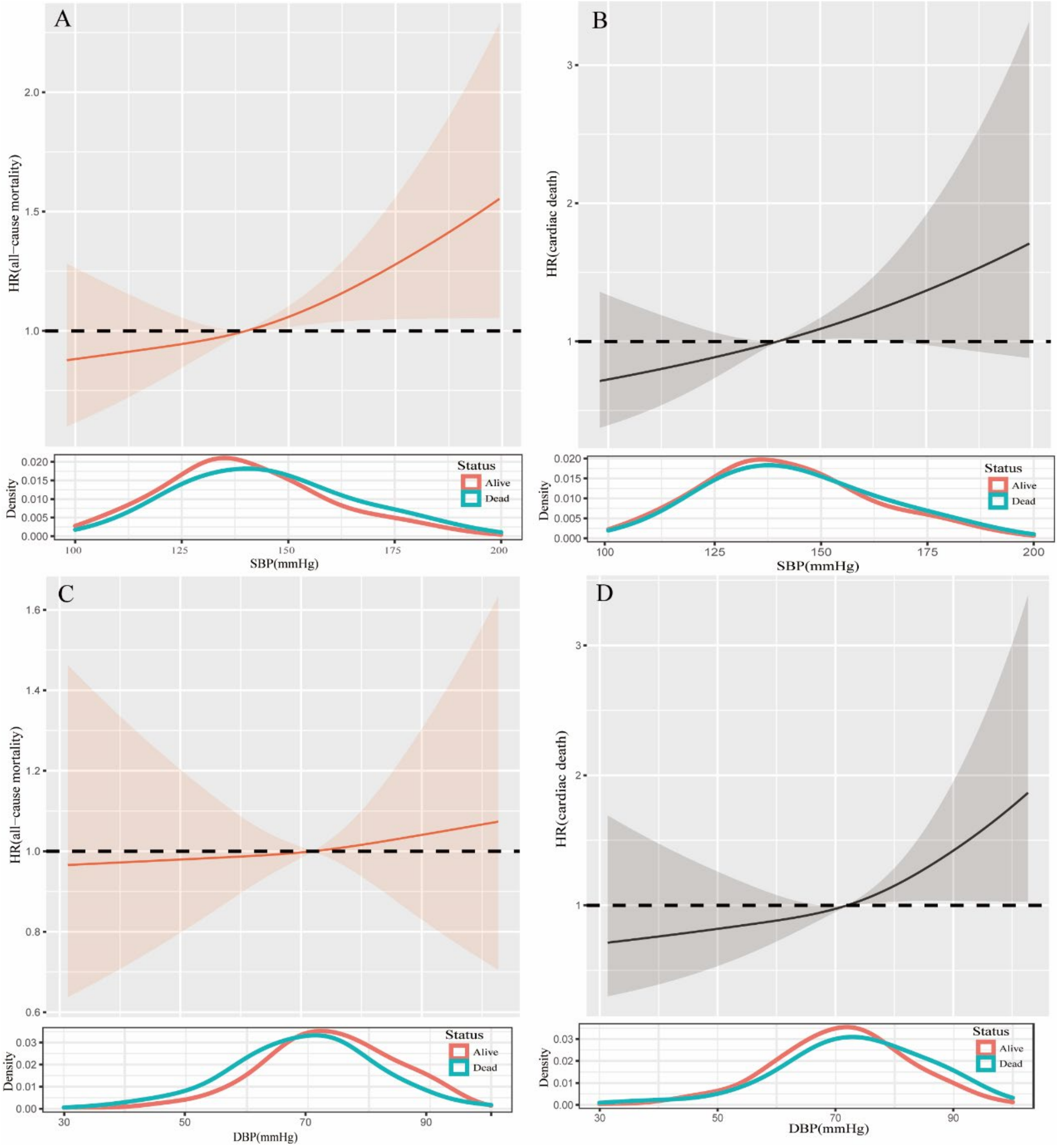

Figure S1. Restricted cubic spline plots of the association between blood pressure and mortality in non-frail participants. A total of 1001 elder non-frail participants were concluded in these analyses. Analyses were adjusted for age, gender, race, diabetes, stroke, coronary artery disease, smoker, and antihypertensive medication. SBP, systolic blood pressure; DBP, diastolic blood pressure; HR, hazard ratio.

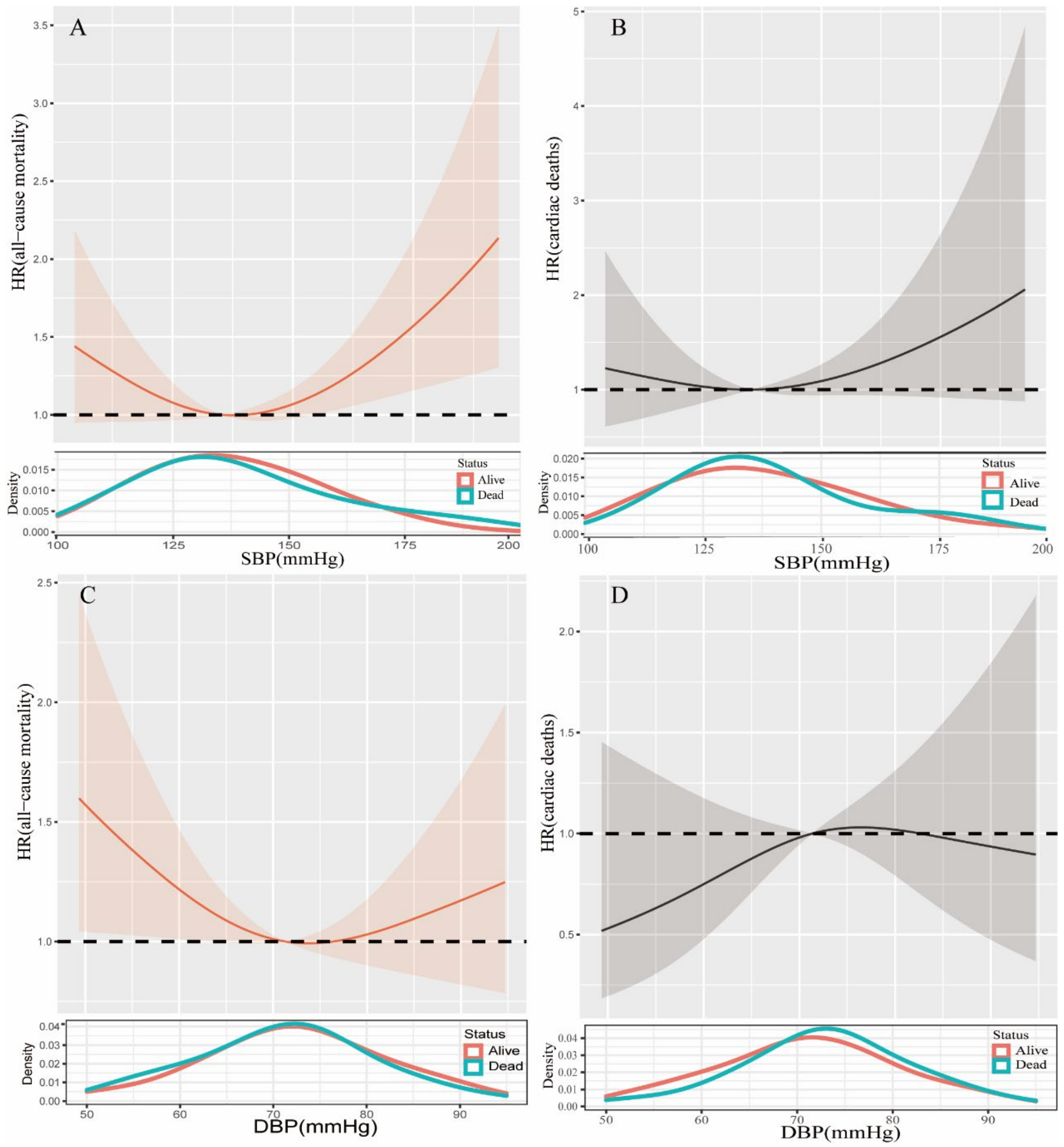

Figure S2. Restricted cubic spline plots of the association between blood pressure and mortality in (pre)frail participants aged<75 years. Analyses were adjusted for age, gender, race, diabetes, stroke, coronary artery disease, smoker, and antihypertensive medication, and frailty. SBP, systolic blood pressure; DBP, diastolic blood pressure; HR, hazard ratio.

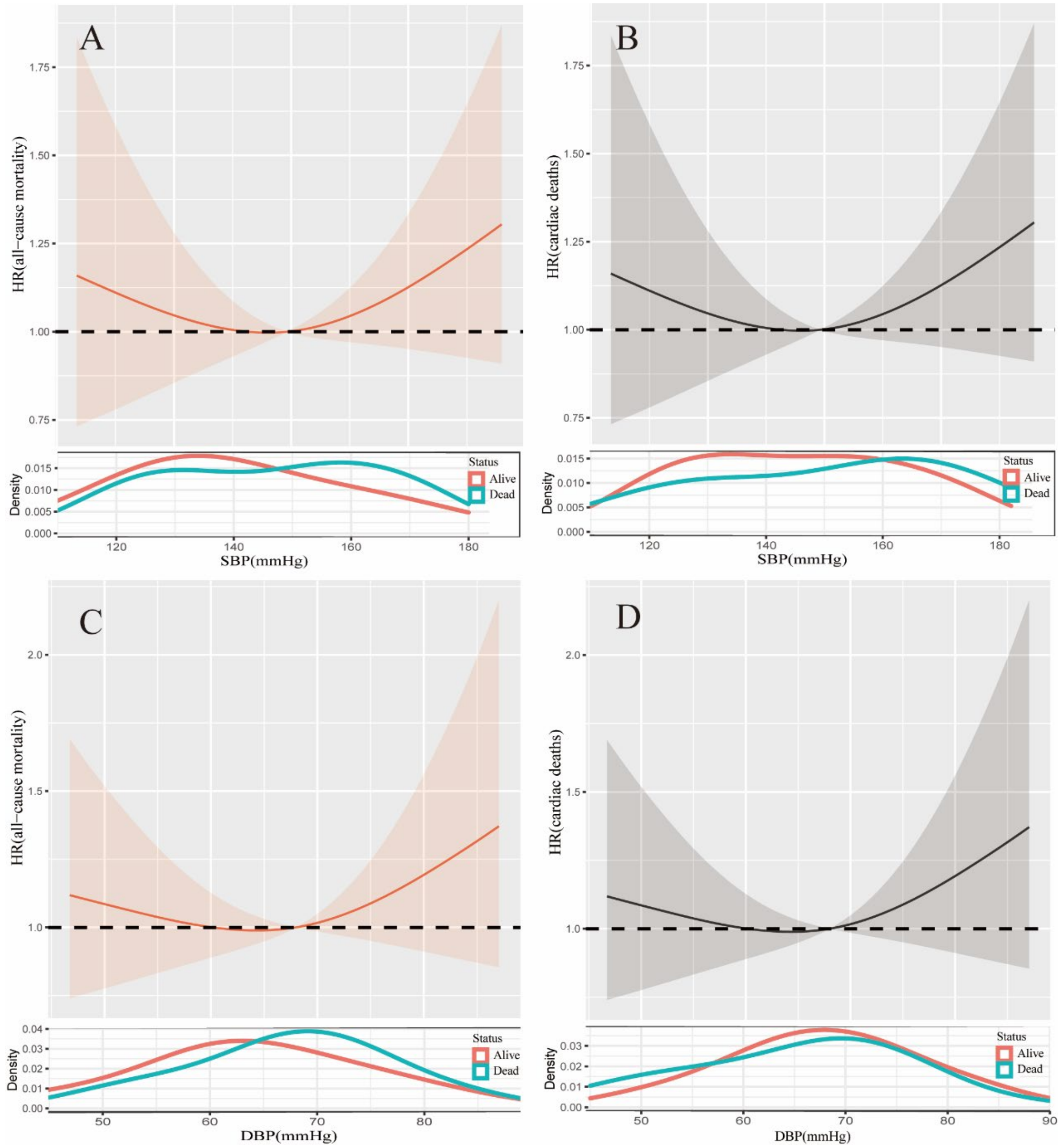

Figure S3. Restricted cubic spline plots of the association between blood pressure and mortality in (pre)frail participants aged≥75 years. Analyses were adjusted for age, gender, race, diabetes, stroke, coronary artery disease, smoker, and antihypertensive medication, and frailty. SBP, systolic blood pressure; DBP, diastolic blood pressure; HR, hazard ratio.
