# Supplementary material for: Effect of blood pressure on the mortality of the elderly population with (pre)frailty: Results from NHANES 1999–2004
Source: Front Cardiovasc Med. 2022 Aug 1;9:919956. doi: 10.3389/fcvm.2022.919956 (PMC9376324; doi:10.3389/fcvm.2022.919956)
Supplement: Supplementary file 2 [file Data_Sheet_2.pdf]

Table S1. Baseline characteristics of the population among frail degree

| Factors | No-frailty (n=1001) | Prefrailty (n=442) | Frailty (n=84) | P-value |
|---|---|---|---|---|
| Age (years), median [Q1-Q3] | 70[64-77] | 70[65-78] | 70[65-78] | 0.204 |
| Male, n (%) | 552 (55.1) | 170 (38.5) | 44 (51.2) | <0.001 |
| Race/ethnicity, n (%) | | | | <0.001 |
| Mexican American | 231 (23.1) | 123 (27.8) | 26 (30.2) | |
| Other Hispanic | 47 (4.7) | 24 (5.4) | 7 (8.1) | |
| Non-Hispanic white | 554 (55.3) | 195 (44.1) | 33 (38.4) | |
| Non-Hispanic Black | 153 (15.3) | 84 (19.0) | 20 (23.3) | |
| Other race | 16 (1.6) | 16 (3.6) | 0 (0) | |
| Education level, n (%) | | | | <0.001 |
| Less than high school | 449 (44.9) | 252 (57.0) | 62 (72.1) | |
| High school | 240 (24.0) | 90 (20.4) | 10 (11.6) | |
| More than high school | 312 (31.2) | 100 (22.6) | 14 (16.3) | |
| SBP (mmHg), median [Q1-Q3] | 140[128-155] | 139[127-158] | 134[118-158] | 0.226 |
| SBP categories, n (%) | | | | 0.004 |
| <110mmHg | 52 (5.2) | 24 (5.4) | 10 (11.6) | |
| 110-130mmHg | 220 (22.0) | 110 (24.9) | 30 (34.9) | |
| 130-150mmHg | 391 (39.1) | 148 (33.5) | 17 (19.8) | |
| 150-170mmHg | 216 (21.6) | 99 (22.4) | 17 (19.8) | |
| ≥170mmHg | 122 (12.2) | 61 (13.8) | 12 (14.0) | |
| DBP (mmHg), median [Q1-Q3] | 72[64-79] | 71[63-77] | 69[63-77] | 0.050 |
| DBP categories, n (%) | | | | 0.128 |
| <60mmHg | 152 (15.2) | 77 (17.4) | 15 (17.4) | |
| 60-70mmHg | 271 (27.1) | 120 (27.1) | 29 (33.7) | |
| 70-80mmHg | 338 (33.8) | 169 (38.2) | 23 (26.7) | |
| 80-90mmHg | 169 (16.9) | 56 (12.7) | 14 (16.3) | |
| ≥90mmHg | 71 (7.1) | 20 (4.5) | 5 (5.8) | |
| Comorbidities, n (%) | | | | |
| Diabetes | 155 (15.5) | 93 (21.0) | 38 (44.2) | <0.001 |
| Hyperlipidemia | 455 (45.5) | 212 (52.0) | 40 (46.5) | 0.677 |
| COPD | 39 (3.9) | 53 (12.0) | 13 (15.1) | <0.001 |
| Stroke | 57 (5.7) | 39 (8.8) | 20 (23.3) | <0.001 |
| CAD | 68 (6.8) | 57 (12.9) | 17 (19.8) | <0.001 |
| Asthma | 79 (7.9) | 62 (14.0) | 9 (10.5) | 0.001 |
| Cancer | 153 (15.3) | 78 (17.6) | 10 (11.6) | 0.292 |
| Thyroid disease | 619 (61.8) | 316 (71.5) | 60 (69.8) | 0.001 |
| CKD | 2 (0.2) | 5 (1.1) | 0 (0) | 0.044 |
| Smoker, n (%) | 508 (50.7) | 234 (52.9) | 53 (61.6) | 0.137 |
| Alcohol user, n (%) | 626 (62.5) | 240 (54.3) | 53 (61.6) | 0.012 |
| Antihypertensive medication, n (%) | 888 (88.7) | 425 (96.2) | 81 (94.2) | <0.001 |
| Mortality, n (%) | | | | |

| | | | | |
|---|---|---|---|---|
| All-cause mortality | 514 (51.3) | 292 (66.1) | 71 (82.6) | <0.001 |
| Cardiac death | 183 (18.3) | 102 (23.1) | 20 (23.3) | 0.080 |

Q1, quartile 1; Q3, quartile 3; COPD, chronic obstructive pulmonary disease; CAD, coronary artery disease; CKD, chronic kidney disease; BMI, body mass index

Table S2. Cox regression analyses of all-cause mortality for SBP in (pre)frail and non-frail people

| Categories | Deaths/ All individuals | Unadjusted model HR (95%CI) | Unadjusted model P-value | Adjusted model HR (95%CI) | Adjusted model P-value |
|---|---|---|---|---|---|
| Age (years) | | **1.09[1.08-1.10]** | **<0.001** | **1.09[1.08-1.11]** | **<0.001** |
| Gender | | | | | |
| Male | 478/766 | Ref. | | Ref. | |
| Female | 399/763 | **0.75[0.65-0.85]** | **<0.001** | **0.68[0.59-0.79]** | **<0.001** |
| Race | | | | | |
| Mexican American | 185/380 | Ref. | | Ref. | |
| Other Hispanic | 31/78 | 0.80[0.54-1.17] | 0.241 | 0.68[0.47-1.01] | 0.056 |
| Non-Hispanic white | 488/782 | **1.45[1.22-1.72]** | **<0.001** | 1.14[0.95-1.37] | 0.160 |
| Non-Hispanic Black | 155/257 | **1.37[1.11-1.70]** | **0.004** | 1.20[0.96-1.49] | 0.105 |
| Other race | 18/32 | 1.22[0.75-1.97] | 0.428 | 0.79[0.48-1.29] | 0.342 |
| Diabetes | | | | | |
| Yes | 192/286 | **1.40[1.20-1.65]** | **<0.001** | **1.45[1.22-1.72]** | **<0.001** |
| No | 685/1243 | **Ref.** | | **Ref.** | |
| Stroke | | | | | |
| Yes | 93/116 | **2.10[1.69-2.61]** | **<0.001** | **1.34[1.07-1.67]** | **0.010** |
| No | 784/1413 | **Ref.** | | Ref. | |
| CAD | | | | | |
| Yes | 105/142 | **1.65[1.34-2.02]** | **<0.001** | 1.21[0.98-1.49] | 0.082 |
| No | 772/1387 | Ref. | | Ref. | |
| Smoker | | | | | |
| Yes | 496/795 | **1.35[1.18-1.54]** | **<0.001** | **1.39[1.20-1.60]** | **<0.001** |
| No | 381/734 | Ref. | | **Ref.** | |
| Frail status | | | | | |
| Non-frailty | 514/1001 | **Ref.** | | **Ref.** | |
| Prefrailty | 292/442 | **1.59[1.38-1.84]** | **<0.001** | **1.70[1.46-1.98]** | **<0.001** |
| Frailty | 71/86 | **3.03[2.36-3.89]** | **<0.001** | **3.20[2.45-4.18]** | **<0.001** |
| Antihypertensive medication | | | | | |
| Yes | 831/1394 | **2.11[1.57-2.84]** | **<0.001** | **1.40[1.03-1.89]** | **0.030** |
| No | 46/135 | Ref. | | Ref. | |
| SBP categories | | | | | |
| <110mmHg | 45/86 | 1.02[0.74-1.40] | 0.907 | 1.05[0.76-1.44] | 0.788 |
| 110-130mmHg | 205/360 | 1.13[0.94-1.35] | 0.186 | 1.07[0.90-1.29] | 0.440 |
| 130-150mmHg | 290/556 | Ref. | | Ref. | |
| 150-170mmHg | 199/332 | **1.27[1.06-1.51]** | **0.011** | 1.04[0.87-1.25] | 0.676 |
| ⩾170mmHg | 138/195 | **1.71[1.40-2.10]** | **<0.001** | **1.46[1.19-1.80]** | **<0.001** |

The analyses were adjusted for age, gender, race, diabetes, stroke, coronary artery disease, smoker, antihypertensive medication, and frail status. SBP, systolic blood pressure; CAD, coronary artery disease; Ref, reference. HR, hazard ratio; CI, confidence interval.

Table S3. Subgroup analyses of the risks of all cause-mortality for SBP.

| Subgroups | SBP: <110mmHg | SBP: 110-130mmHg | SBP: 130-150mmHg | SBP: 150-170mmHg | SBP: ⩾170mmHg |
|---|---|---|---|---|---|
| Frail status | | | | | |
| Frailty | 1.32 (0.50-3.47) | 0.78 (0.36-1.66) | Ref. | 0.92 (0.41-2.08) | 1.50 (0.62-3.65) |
| Prefrailty | 1.73 (1.00-2.99)* | 1.05 (0.76-1.44) | Ref. | 1.10 (0.79-1.52) | 1.63 (1.12-2.37)* |
| Age | | | | | |
| Age<75 | 1.00 (0.58-1.73) | 0.89 (0.62-1.29) | Ref. | 0.77 (0.49-1.22) | 1.89 (1.17-3.06) |
| Age≥75 | 3.06 (1.28-7.29)* | 1.13 (0.70-1.83) | Ref. | 1.32 (0.84-2.06) | 1.91 (1.13-3.23)* |

The analyses were adjusted for age, gender, race, diabetes, stroke, coronary artery disease, smoker, antihypertensive medication, and frailty. SBP, systolic blood pressure; *,p<0.05

Table S4. Subgroup analyses of the risks of cardiac death for SBP.

| Subgroups | SBP: <110mmHg | SBP: 110-130mmHg | SBP: 130-150mmHg | SBP: 150-170mmHg | SBP: ⩾170mmhg |
|---|---|---|---|---|---|
| Frail status | | | | | |
| Frailty | 5.97 (0.41-86.44) | 3.88 (0.38-39.63) | Ref. | 2.10 (0.20-21.78) | 13.03 (0.95-179.51) |
| Prefrailty | 1.37 (0.52-3.59) | 0.92 (0.53-1.59) | Ref. | 1.04 (0.60-1.82) | 1.64 (0.85-3.15) |
| Age | | | | | |
| Age<75 | 0.68 (0.23-1.97) | 0.96 (0.53-1.74) | Ref. | 0.60 (0.29-1.27) | 2.35 (1.07-5.16)* |
| Age≥75 | 10.34 (2.13-50.06) | 1.91 (0.62-5.91) | Ref. | 3.14 (1.07-9.22) | 4.59 (1.43-14.81) |

The analyses were adjusted for age, gender, race, diabetes, stroke, coronary artery disease, smoker, antihypertensive medication, and frailty. SBP, systolic blood pressure; *,p<0.05
